# Comparative Systematic Analysis of Gray Matter Biophysical Models on a Public Dataset

**DOI:** 10.1002/mrm.70507

**Published:** 2026-07-16

**Authors:** Santiago Mezzano, Quentin Uhl, Tommaso Pavan, Jasmine Nguyen‐Duc, Hansol Lee, Susie Huang, Ileana Jelescu

**Affiliations:** ^1^ Department of Radiology Lausanne University Hospital (CHUV) Lausanne Switzerland; ^2^ Faculty of Biology and Medicine University of Lausanne Lausanne Switzerland; ^3^ School of Psychology University of Auckland Auckland New Zealand; ^4^ Athinoula A. Martinos Center for Biomedical Imaging Charlestown Massachusetts USA; ^5^ Department of Radiology, Massachusetts General Hospital Harvard Medical School Boston Massachusetts USA

**Keywords:** biophysical modeling, diffusion MRI, gray matter microstructure, NEXI

## Abstract

**Purpose:**

Biophysical models of diffusion tailored to characterize gray matter (GM) microstructure are gaining traction in the neuroimaging community. NEXI, SMEX, SANDI, and SANDIX represent recent efforts to account for different microstructural features, such as soma contributions and inter‐compartment exchange, in the diffusion MRI (dMRI) signal. The purpose of this work is to provide a comparative evaluation of these four GM diffusion models.

**Methods:**

A comparative analysis of NEXI, SMEX, SANDI, and SANDIX was performed using a single, publicly available in vivo human dataset, the Connectome Diffusion Microstructure Dataset (CDMD), acquired with two diffusion times. Cortical microstructure metrics were estimated in 26 healthy subjects using the open‐source Gray Matter Swiss Knife toolbox, and goodness of fit, anatomical patterns, and consistency with previous studies were evaluated.

**Results:**

CDMD data yielded GM parameter estimates consistent with values reported in previous studies across all four models. NEXI and SMEX produced similar cortical anatomical patterns, with consistent regional distributions across diffusion times. Goodness of fit varied across models, with NEXI showing the best fit, followed by SMEX, and finally SANDIX. SANDI parameter estimates showed strong dependence on diffusion‐time selection and fitting algorithm.

**Conclusion:**

This retrospective cross‐model analysis establishes the feasibility of estimating exchange models from only two diffusion times and highlights trade‐offs in biological specificity, model complexity, and fitting robustness, which are critical considerations when selecting a model for future clinical and research applications.

## 1 Introduction

Diffusion‐weighted magnetic resonance imaging (dMRI) is at the forefront of neuroimaging technologies, offering a unique window into the microstructural intricacies of brain tissue and cellular structures in vivo, non‐invasively [1]. dMRI exploits the micrometer‐scale movement of water molecules, whose diffusion patterns are influenced by cellular geometry, membrane permeability, and the presence of intracellular organelles at comparable scales [2, 3]. This holds promise for both advancing our understanding of brain structure and enabling the early detection of neurological and psychiatric disorders [4, 5].

The sensitivity of the dMRI signal to microstructure is indirect, and cellular parameters can be estimated through biophysical modeling which links the measured MR signal to the underlying neurobiological microstructure properties [6]. Biophysical models are, however, tissue specific.

In recent years, the dMRI community has taken up the challenge to develop and validate novel biophysical models tailored to brain gray matter (GM), to account for its specificities in the form of a permeable neurite membrane [7, 8] (unlike myelinated impermeable axons in white matter) or a non‐negligible density of cell bodies [9].

By quantifying the microstructural features of the human cortex in vivo, and interpreting their neurobiological signature, GM biophysical models hold the promise to significantly advance our capabilities in both research and clinical settings.

This comparative analysis will thus focus on four biophysical models of GM, all implemented as part of an open‐source tool named Gray Matter Swiss Knife. The GM models under consideration are Neurite Exchange Imaging (NEXI) [7], Standard Model with EXchange (SMEX) [8], Soma and Neurite Density Imaging (SANDI) [9], and Soma And Neurite Density Imaging with Exchange (SANDIX) [8]. As these models make their way toward translation to clinical MRI scanners [10, 11], it is important to test their performance on publicly available datasets, more of which will hopefully be acquired and shared in the years to come. Here, we performed a retrospective analysis on 26 healthy subjects from the MGH Connectome Diffusion Microstructure open‐source Dataset (CDMD) [12], leveraging its ideal acquisition parameters of multi‐diffusion times and high gradient strengths required for estimating these GM models [7, 8, 9].

The goal of this study is to: (i) evaluate the feasibility and reliability of estimating each of the four models on a diffusion protocol comprising two diffusion times and multiple shells, (ii) quantify and compare cortical Region of Interest (ROI) values and surface maps with previous literature on these models based on dedicated and more comprehensive datasets. Moreover, NEXI has only been estimated so far in the human brain using dMRI protocols with three [13] or four [11, 14] diffusion times. The feasibility of NEXI/SMEX estimation based on two diffusion times, previously shown in ex vivo mouse brain [8], would lower the minimum acquisition time and thus increase its potential for clinical translation.

## 2 Theory

**FIGURE 1:**
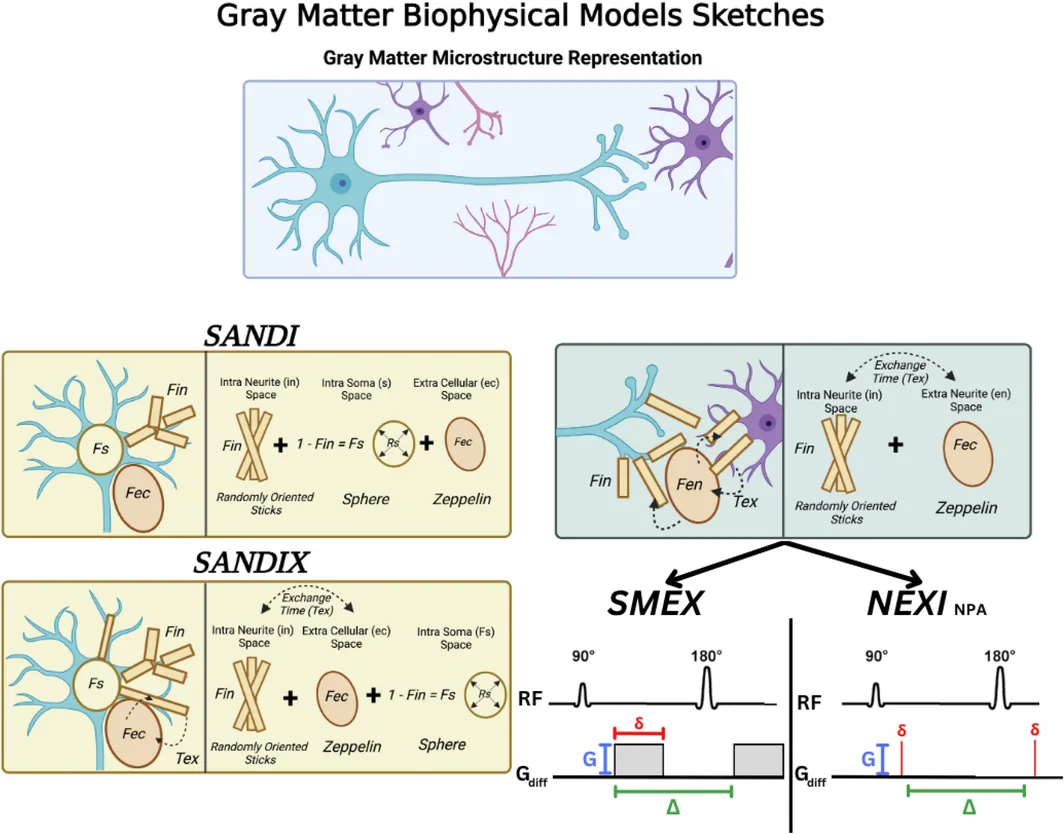
Graphical summary of four gray matter biophysical models—NEXI, SMEX, SANDI, and SANDIX—used to model the diffusion MRI (dMRI) signal in the cortex. Each model includes different compartments to represent intra‐neurite space, intra‐soma space, extra‐neurite space and water exchange mechanisms. For NEXI and SMEX we illustrate the corresponding PGSE (Pulsed Gradient Spin Echo) sequence which highlights their assumptions about diffusion time ($t_{d}$), with SMEX accounting for the gradient duration δ and NEXI incorporating the correction $t_{d}=\Delta -\delta /3$ to account for finite gradient durations. $f_{\text{neurite}}$ ($F_{\text{in}}$), intra‐neurite signal fraction; $f_{\text{extracellular}}$ ($F_{\mathrm{ec}}$), extra‐neurite signal fraction; $f_{\text{soma}}$ ($F_{s}$), intra Soma Space; $R_{\text{soma}}$ ($R_{s}$), apparent soma radius; $t_{\mathrm{ex}}$, exchange time. *Source:* Created in BioRender, Mezzano [53], https://BioRender.com/q54w840.

Here we briefly describe the assumptions behind each of the GM models under consideration (Figure 1). More details can be found in the original papers introducing them [7, 8, 9].

### 2.1 Incorporating the Soma Compartment—SANDI

SANDI [9] is a biophysical model that introduces a three‐compartment framework representing:

- **Neurites:** Randomly oriented sticks with uni‐dimensional intra‐stick axial diffusivity ($D_{i}$) and a relative signal fraction (f).
- **Soma:** Spheres with an apparent radius ($R_{s}$), fixed intra‐sphere diffusivity ($D_{s}$), and a relative signal fraction ($f_{s}$). Diffusion in spheres relies on the Gaussian Phase Approximation.
- **Extracellular Space:** Modeled with Gaussian isotropic diffusion ($D_{e}$) and a relative signal fraction ($1-f-f_{s}$).

SANDI thus allows for the independent estimation of five parameters: neurite signal fraction (f), soma signal fraction ($f_{s}$), intra‐stick diffusivity ($D_{i}$), extracellular diffusivity ($D_{e}$), and soma radius ($R_{s}$) (Figure 1).

This model does not account for inter‐compartment exchange, hence the need to use a protocol with short diffusion time (Δ≤20 ms) to keep the assumption of impermeable stick compartments valid. High b‐values (e.g., above $b=6\;\mathrm{ms}/{\mathrm{\mu m}}^{2}$) within short diffusion times can only be acquired by systems with ultra‐strong gradients, such as preclinical scanners or new generation dedicated human scanners (Connectom 1.0, Connectom 2.0, Magnus). Longer diffusion times would cause the exchange between compartments to contaminate the signal and affect the accuracy of the estimated parameters.

Recent studies have tested the effectiveness of SANDI in multiple contexts. Initially, the model was shown to produce soma and neurite signal fraction maps using multi‐shell sequences with *b*‐values up to 10 $\mathrm{ms}/{\mathrm{\mu m}}^{2}$ on a human Connectom scanner, which agreed with histological images of brain architecture [9]. Additionally, parameter stability was confirmed in in vivo healthy mouse brains, with strong correlations between SANDI‐derived soma signal fractions and histology of cell density data from the Allen Brain Atlas [15]. Recent research reported SANDI estimates across the GM cortical ribbon in the human brain using a 3 T scanner with weaker gradients and a lower maximum *b*‐value of 6 ms/mm^2^ [10], albeit at a longer diffusion time (Δ=39.07 ms), which likely violates the impermeability assumption.

### 2.2 Accounting for Exchange—NEXI, SMEX

Simulations and experimental data have consistently shown that water exchange between neurites and the extracellular space in GM is non‐negligible over typical diffusion times of MRI experiments (20–100 ms) as most neurites are unmyelinated and thus permeable [7, 8, 16, 17].

To address this challenge, two signal models were proposed in parallel, NEXI [7, 14] and the SMEX [8, 11], which are based on the same underlying microstructure but differ in their treatment of the diffusion sequence: NEXI assumes the narrow pulse approximation (NPA), whereas SMEX does not. By incorporating an exchange parameter, NEXI and SMEX eliminate the requirement for short diffusion times and can thus be implemented on clinical‐grade scanners. Both models are parametrized by four parameters to be estimated (Figure 1).

- **Neurites:** Same as SANDI; modeled as a collection of randomly oriented sticks, with intra‐stick uniaxial diffusivity ($D_{i}$) and a relative signal fraction (f).
- **Extracellular Space:** Same as SANDI; considered Gaussian isotropic with characteristic diffusivity ($D_{e}$).
- **Exchange Process:** Exchange between neurites and extracellular space with a characteristic exchange time ($t_{\mathrm{ex}}$).

The signal from the soma is not explicitly considered in NEXI or SMEX and is assumed to be pooled with the extra‐cellular space [7].

While NEXI assumes short gradient pulse durations δ under the NPA, SMEX additionally accounts for finite, and potentially long, pulse durations, making it ideally suited for realistic acquisition protocols on clinical systems. Indeed, NEXI, based on the Kärger model approximation [18], simplifies exchange dynamics by modeling the Pulsed Gradient Spin Echo (PGSE) diffusion encoding pulses as instantaneous Dirac pulses separated by a duration Δ. This assumption allows a more straightforward solution, at the cost of some accuracy. Note however that defining the diffusion time as $t_{d}=\Delta -\delta /3$ allows for some correction for the finite pulse duration δ. SMEX on the other hand uses numerical integration and accounts for dynamics during the diffusion gradient pulse as well.

One requirement shared by both NEXI and SMEX is that of a protocol that samples multiple diffusion times $t_{d}$ or Δ in order to robustly estimate exchange. This requirement substantially increases the acquisition time.

NEXI has been tested on both preclinical and human scanners to quantify GM microstructure [7, 14]. The first NEXI study in the human cortex found very good scan‐rescan reproducibility while maintaining subject‐specific GM variability [14]. This work also emphasized the importance of accounting for the Rician noise floor when fitting magnitude data, which was added to the model termed NEXI_RM_ (for Rician Mean).

SMEX was initially implemented and tested on preclinical data in ex vivo mouse cortex [8], and was later applied in the mouse brain in vivo [19]. Recently, the performance of SMEX versus NEXI was also demonstrated on a clinical scanner with longer gradient pulses [11].

Throughout this manuscript, we will reference neurite signal fraction as f for consistency across all four models and other works. While SANDI implementations in other studies distinguish between neurite ($f_{n}$) and soma ($f_{s}$) fractions, NEXI and SMEX do not explicitly model soma. Therefore, for a uniform comparative framework, we use f to represent neurite fraction in all models, with $f_{s}$ used for soma fraction in SANDI and SANDIX.

### 2.3 Accounting for Soma and Exchange—SANDIX

A combination of SANDI and NEXI is SANDIX [8]. SANDIX accounts for an impermeable soma compartment in addition to the exchanging neurites and extra‐cellular space. This results in a three‐compartment model with six free parameters: f, $f_{s}$, $R_{s}$, $D_{i}$, $D_{e}$, and $t_{\mathrm{ex}}$ (Figure 1). The feasibility of estimating so many parameters has only been tested on an extensive ex vivo preclinical dataset [8], and has yet to be evaluated in the human cortex.

As mentioned, all previous human in vivo studies estimated NEXI from data parsing four diffusion times. SMEX and SANDIX were previously estimated in the mouse cortex using two diffusion times on a pre‐clinical scanner [8]. The MGH CDMD [12] used in the current study covers a larger cohort size than previous NEXI/SMEX datasets, and samples only two diffusion times, making it the first instance where these models are applied with this type of reduced acquisition in the human cortex. In this context, the value of this dataset lies in the fact that the acquisition protocol was not optimized for any specific model, allowing for an unbiased comparison across modeling frameworks.

## 3 Methods

We performed a retrospective analysis on 26 healthy young subjects downloaded from the CDMD [12], to compare cortical microstructure estimates stemming from each of the GM models. We also conducted a post hoc analysis comparing NEXI and SMEX to elucidate further differences in quality of fit estimates and model behavior in this dataset.

### 3.1 Participants

The study protocol was approved by the institutional review board of Mass General Brigham, following compliance with all relevant ethical regulations. Written informed consent was obtained from all participants. Data were acquired from 26 healthy, cognitively normal adults (36.8 ± 14.6 years old, 17 females).

### 3.2 Connectome Diffusion Microstructure Dataset (CDMD)

Detailed methods can be found in [12]. Briefly, data were acquired on a 3 T Connectom MRI scanner, with a gradient coil of 300 mT/m. For anatomical reference, T1w images were acquired using a multi‐echo magnetization‐prepared gradient echo (MEMPRAGE) sequence, at 1‐mm isotropic resolution. Diffusion‐weighted images were acquired using a single‐refocused pulsed‐gradient spin‐echo echo‐planar imaging (EPI) sequence, with b‐values ranging from 0.05 to 6 ms/mm^2^ at Δ=19ms and from 0.2 to 17.8 ms/mm^2^ at Δ=49ms, in 16 increments. This included 32 or 64 diffusion encoding directions depending on the b‐value, alongside 50 non‐diffusion‐weighted (b=0) images. Other fixed parameters were gradient duration δ of 8 ms, TE/TR of 77 ms/3.8 s, at 2‐mm isotropic resolution. The total scan time for the diffusion MRI protocol was 55 min. Downloaded diffusion data were already pre‐processed with a standard pipeline, correcting for gradient nonlinearity, susceptibility and eddy current‐induced image distortions, and motion [12]. The full acquisition protocol can be found in the Supporting Information (Figure S1).

Importantly, for a subset of 14 participants, phase images in addition to magnitude images were available. From complex‐valued data, real‐valued diffusion data were extracted after filtering the phase, as described by [12]. This method preserved the Gaussian noise distribution in the data which better aligns with the assumptions underlying MP‐PCA denoising. Additionally, the elevated Rician noise floor at high *b*‐values in the magnitude data was largely attenuated in the real‐valued data. Removing or accounting for the Rician noise floor has been shown to be critical when processing GM biophysical models, particularly when estimating parameters related to exchange between compartments [14].

Here, we processed both magnitude data from 26 subjects as well as real‐valued data from 14 subjects to investigate accuracy in parameter estimates within and between biophysical models across varying de‐noising strategies. However, a more thorough analysis and comparison of models was done on the 26 subjects with magnitude data, leveraging the increased number of subjects and the more typical context of magnitude images in clinical settings.

### 3.3 Gray Matter Swiss Knife

Gray Matter Swiss Knife (https://github.com/QuentinUhl/graymatter_swissknife) is an open‐source python package that implements the estimation of various parameter maps from four different GM biophysical models (SMEX, NEXI, SANDI, SANDIX). Gray Matter Swiss Knife uses nonlinear least squares (NLS) with initial grid search, utilizing the L‐BFGS‐B algorithm and minimize function from the scipy library in Python [20].

For datasets where both real‐valued and magnitude data were available (*N* = 14), all four models (NEXI, SMEX, SANDI, and SANDIX) were estimated without Rician mean correction in real‐valued data, and with Rician mean correction (RM) in the magnitude data.

For all magnitude datasets (*N* = 26), the four models were estimated. The noise map calculated from MP‐PCA denoising at high SNR (low b‐values) was used to correct for Rician Mean [14] in NEXI, SMEX, SANDIX and SANDI.

As SANDI assumes no exchange between compartments, it is not designed to be applied to multiple diffusion time data. Therefore, SANDI model estimates were calculated for the shorter diffusion time Δ=19 ms only. This single‐diffusion‐time approach aligns with the model's theoretical assumptions and evaluates SANDI performance under its intended conditions, when the exchange signature is minimized.

### 3.4 Cortical Parcellation, Volumetric Maps and Surface Maps

GM ROIs from the Desikan–Killiany–Tourville (DKT) atlas [21] were segmented on the anatomical MEMPRAGE image using FastSurfer [22] and transformed into native diffusion space using linear registration of distortion‐corrected b = 0 images to T1 images. The cortical ribbon was segmented by merging the GM ROIs obtained from the DKT segmentation. Mean parameter estimates were calculated by finding the median in every DKT ROI and then calculating the mean from all ROIs in the cortical ribbon. Calculating the median as opposed to the mean at the ROI level reduces the impact of outliers, which can arise due to noise, artifacts, partial volume or segmentation errors. It also reproduces the same analysis that other papers have used to quantify average GM microstructure [10, 14].

To visually inspect the parameter maps generated for each biophysical model, average maps from the 26 subjects were generated in standard space (MNI) [23].

Moreover, SMEX, NEXI parameter maps were transformed into Freesurfer's fsaverage subject space and projected onto cortical surfaces to visualize and compare anatomical trends with those presented in previous works [11, 14]. One subject was discarded from surface map reconstruction due to an incomplete cortical coverage within the field of view in its CDMD dataset.

An in depth comparison of SANDI and SANDIX parameter map comparisons including surface maps derived from this same CDMD dataset have recently been reported [24] and were therefore not reported in this paper.

### 3.5 Quality of Fit

In order to compare the quality of the fit of the model on the measured data, we compared the Measured vs. Fitted Signal as a function of $\sqrt{b}$ at different diffusion times, and also computed the corrected Akaike Information Criterion (AICc) [25] for each model. This metric evaluates the goodness of fit while penalizing model complexity, aiming to achieve a balance between accuracy and simplicity.

### 3.6 Simulations

We performed simulations to evaluate the accuracy and robustness of SMEX and NEXI parameter estimates against known ground truth values from two diffusion times. Synthetic SMEX signals were generated using the same values of δ, diffusion times, and b‐values as in the experimental acquisition. We used SMEX to generate the ground truth signals because this model explicitly accounts for the finite diffusion gradient pulse width as opposed to NPA from NEXI.

The ground truth parameters for each signal were randomly sampled from the combined NEXI and SMEX experimental parameter estimates, under the constraint that $D_{i}>D_{e}$ [26, 27, 28].

For each powder‐averaged signal at a given b–$t_{d}$ combination, we generated a number of Rician noise realizations equal to the number of diffusion directions acquired for that specific b–δ, for each ground truth instance. The signal‐to‐noise ratio (SNR) for each ground truth instance was randomly drawn from the in vivo SNR distribution at b = 0.

The noisy signals were then averaged to emulate powder‐averaged magnitude images, which improves the SNR but does not reduce the Rician noise floor. In total, a dataset of 10′000 ground truth parameter combinations was constructed using this approach.

The generated signals were then fitted using both the SMEX and NEXI models to assess error propagation when the ground truth perfectly follows the SMEX model. The discrepancy between the ground truth and the estimated parameters was quantified.

## 4 Results

### 4.1 Quantitative Comparison Across All GM Biophysical Models

#### 4.1.1 Summary of Parameter Estimates

**FIGURE 2:**
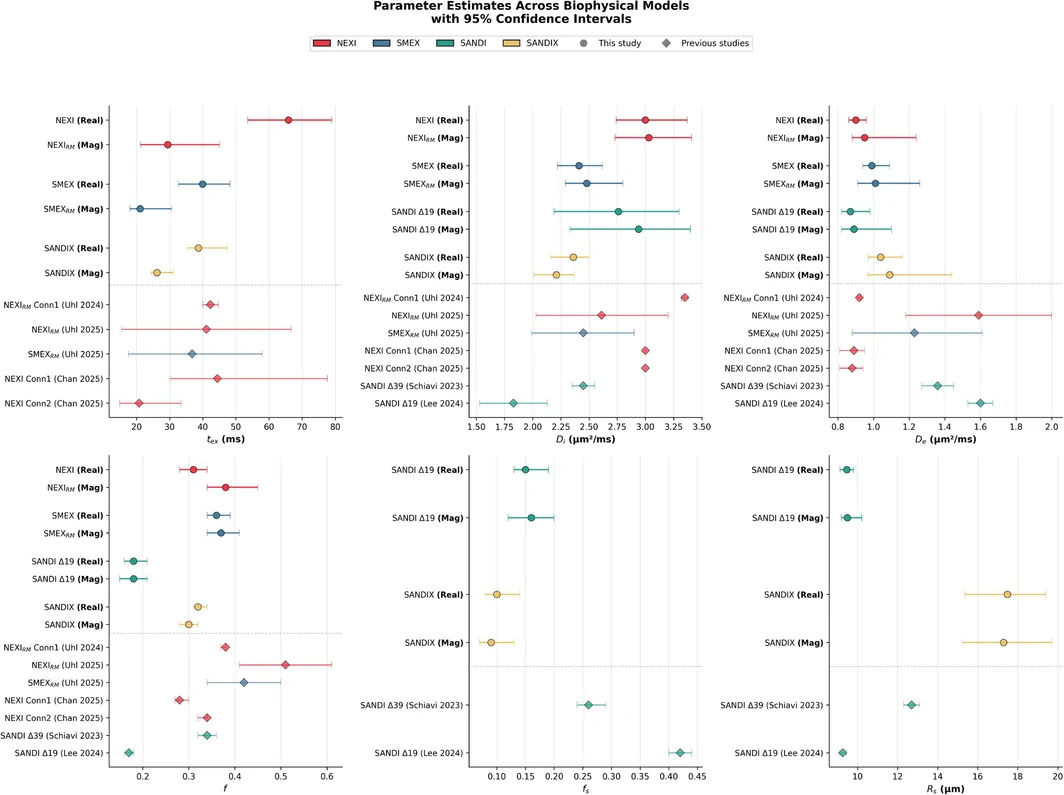
Mean parameter estimates and ranges across biophysical models applied to the cortical ribbon. Circles denote results from the present study, with models fitted to both real‐valued data (*N* = 14 subjects) and magnitude data (*N* = 26 subjects). Diamonds denote values reported in previous studies. Colors indicate model family: NEXI (red), SMEX (blue), SANDI (green), and SANDIX (yellow). The dashed horizontal line separates the present results from previous literature. SANDI was fitted using Δ=19 ms only (SANDI Δ19). Parameters shown are exchange time ($t_{\mathrm{ex}}$), intra‐neurite diffusivity ($D_{i}$), extra‐cellular diffusivity ($D_{e}$), neurite signal fraction (f), soma signal fraction ($f_{s}$), and soma radius ($R_{s}$).

In Figure 2 we present parameter estimates from the cortical ribbon derived from the real‐valued data of 14 subjects and the magnitude data of 26 subjects along with their 95% confidence intervals. In addition, we provide mean parameter estimates from five previous studies examining cortical ribbon metrics derived from SANDI [10, 29] and NEXI/SMEX [11, 13, 14] for comparison. Detailed numerical values are provided in Supporting Information (Table S1).

Figure 2 shows that parameter estimates across models are fairly consistent between denoising strategies (complex vs. magnitude denoising) and with previous literature reports using other datasets. Nevertheless, within the CDMD dataset, the parameter that varies the most between real‐ vs. magnitude data is the exchange time ($t_{\mathrm{ex}}$), with consistently higher values in real‐valued vs. magnitude data ($t_{\mathrm{ex}}=68.8$ ms vs. 29.3 ms for NEXI, and $t_{\mathrm{ex}}=41.4$ ms vs. 21.1 ms for SMEX). SANDIX also yielded similar differences between real‐valued ($t_{\mathrm{ex}}=40.1$ ms) and magnitude ($t_{\mathrm{ex}}=25.9$ ms) data. All NEXI estimates on real‐valued data yielded similar cortical $t_{\mathrm{ex}}$ between 40 and 70 ms, which is aligned with previous Connectom 1 estimates [14] but less with recent Connectom 2 results [13].

For intra‐cellular diffusivity, all models show similar values for real and magnitude data. The NEXI and SANDI estimates are higher than SMEX and SANDIX, consistent with previous implementations [11, 14]. Notably, $D_{i}$ was the noisiest of all estimates, frequently hitting the upper bound of the range as shown in Figure S2. The challenge in estimating $D_{i}$ with good accuracy and precision is also reported in previous NEXI and SANDI works [7, 9, 11, 13, 14, 30].

NEXI and SMEX cortical extracellular diffusivity values are comparable between different denoising strategies as well as previous implementations using Connectom Scanners ($D_{e}=0.9-1$ ${\mathrm{\mu m}}^{2}$/ms). However, they seem to increase when using clinical scanners [11]. The same trend was found with SANDI, which overall reported $D_{e}\approx 0.8-0.9$ ${\mathrm{\mu m}}^{2}$/ms for both real and magnitude data, also lower than previously reported in clinical data using a single, longer diffusion time (1.36 ${\mathrm{\mu m}}^{2}$/ms [10]).

All models showed consistent neurite signal fractions ranging 20%–40%, also aligned with previous literature. SANDI Δ=19ms is notably the model which estimates the lowest f = 0.18 in the CDMD dataset.

For Soma Fraction, SANDI Δ=19ms yielded $f_{s}\approx 0.15$–0.16, lower than previously reported values of $f_{s}=0.26$ [10] and $f_{s}=0.42$ [29], both of which employed Random Forest (RF) fitting. Similarly, SANDI yielded higher $D_{i}$ estimates ($\approx 2.8\;{\mathrm{\mu m}}^{2}/\mathrm{ms}$) compared to Lee et al. ($D_{i}=1.83\;{\mathrm{\mu m}}^{2}/\mathrm{ms}$) [29]. Notably, Lee et al. [29] used an identical acquisition protocol to the CDMD when restricting SANDI fitting to Δ=19 ms, yet RF algorithm yielded substantially different $f_{s}$ and $D_{i}$, suggesting that these discrepancies arise primarily from the fitting framework (RF regression [29] versus NLS) rather than the data.

#### 4.1.2 Quality of Fit

**FIGURE 3:**
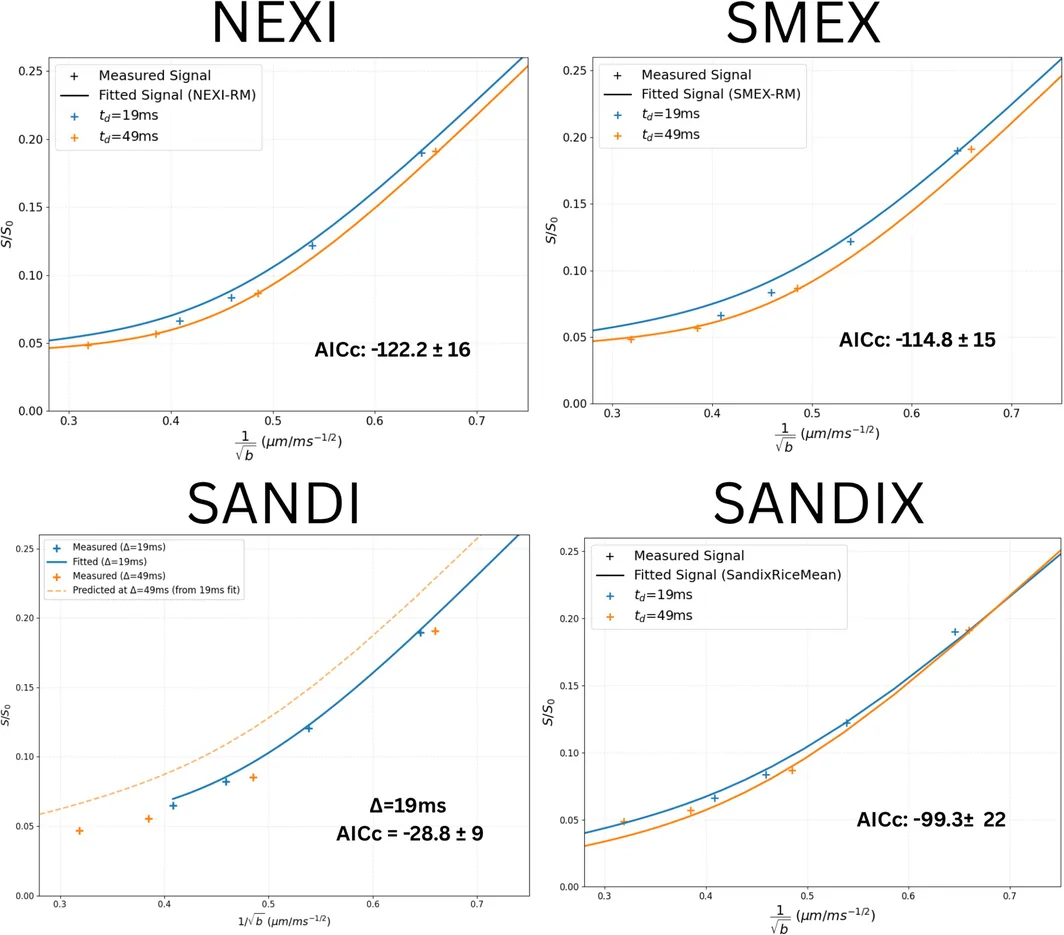
Measured vs. model‐predicted signal decay across cortical ribbon voxels as a function of $\sqrt{b}$ at diffusion times of 19 ms and 49 ms. Results are shown for SMEX, NEXI, SANDI, and SANDIX applied to magnitude data for all 26 subjects. Dots represent experimental signal and curves show voxel‐wise model predictions averaged across the cortical ribbon. The figure highlights model accuracy and differences across diffusion times, b‐values, and data types. Goodness of fit was assessed using AICc, with lower values indicating better fit. While 16 b‐values were acquired in total, the plots display model fits and measured signal for the upper range 2.3 to $13.5\;\mathrm{ms}/{\mathrm{\mu m}}^{2}$. For SANDI, parameters fitted at Δ=19 ms (blue) are used to predict the signal at Δ=49 ms (dashed orange).

Figure 3 compares measured experimental data (average cortical ribbon signal) with the estimated fits from GM biophysical models. Fits are performed voxel‐wise, and the resulting predicted curves are averaged across cortical ribbon voxels.

Overall, the quality of fit illustrates a clear ranking of model performance: NEXI achieved the best fit (AICc = −122.2±16), followed by SMEX (AICc = −114.8±15), and finally SANDIX (AICc = −99.3±22). The good performance of NEXI is expected in a short gradient pulse configuration (δ = 8 ms). Interestingly, despite SMEX's more complete treatment of finite pulse effects, it yields poorer fits than NEXI for this dataset.

Nevertheless, a better fit does not always guarantee a more accurate representation of the underlying parameters, and the quality of the fit might be influenced by voxels in the cortical ribbon with partial volume effects.

The examination of voxel signal distributions in the cortical ribbon (Figure S2) reveals that SMEX mitigates extreme values and attenuates the pronounced multi‐modal distribution observed for $t_{\mathrm{ex}}$ and f with NEXI. It also yields fewer voxels reaching the upper bound constraints compared to NEXI.

SANDI was not designed for fitting across multiple diffusion times; therefore, SANDI parameters were estimated using only the shortest diffusion time (Δ=19 ms). The lower AICc value for SANDI (−28.8±9) is not directly comparable to other models, as it is calculated from fewer data points (8 b‐values from the 19 ms diffusion time versus 16 b‐values across both diffusion times for the other models). To illustrate SANDI's inability to properly capture time‐dependence across diffusion times, the dashed orange curves in Figure 3 show SANDI predictions at Δ=49 ms based on parameters estimated at Δ=19 ms. The signal dependence predicted by SANDI is inverted compared to the measured signal, as SANDI predicts higher signal at longer diffusion times, whereas the experimental data shows lower signal.

SANDIX, despite including a soma compartment, showed poorer fit than NEXI and SMEX, likely due to the increased number of parameters requiring estimation, which compromises precision.

The comparison between real‐valued and magnitude data fits is provided in Supporting Information (Figure S3). Across NEXI, SMEX, and SANDIX, magnitude data processed with Rician mean correction yielded lower AICc scores than real‐valued data, indicating improved fit quality. This improvement likely reflects the incomplete removal of noise bias by the denoising method applied to real‐valued data. However, SANDI estimates at both single (Δ=19 ms) and multiple diffusion times showed minimal differences in quality of fit between real‐valued and magnitude data, as well as their cortical ribbon parameter estimates (Figure 2).

#### 4.1.3 Group Average Parametric Maps for All Models

The group average parametric maps confirm the trends outlined in the forest plot analysis in Figure 2 and the goodness of fit in Figure 3: SANDIX maps are the noisiest across models, and $D_{i}$ maps are the noisiest across model parameters. In SANDI, soma fraction $f_{s}$ and radius $R_{s}$ also display limited precision, underscoring the challenge in estimating soma alongside neurite and extracellular compartments.

Neurite density (f) shows the expected higher fraction in WM versus GM. In those models that consider exchange, the exchange time is also longer in WM than GM, consistent with a higher density of myelinated “impermeable” axons and thus a slower inter‐compartment exchange. Notably, SANDI fitted at Δ=19 ms using NLS does not display the expected anatomical gray‐white matter contrast in soma fraction ($f_{s}$), which has been consistently reported in previous studies, which all used RF implementation [24, 29, 31]. This discrepancy further underscores that soma‐related parameter estimates are sensitive to the fitting framework (NLS versus RF).

**FIGURE 4:**
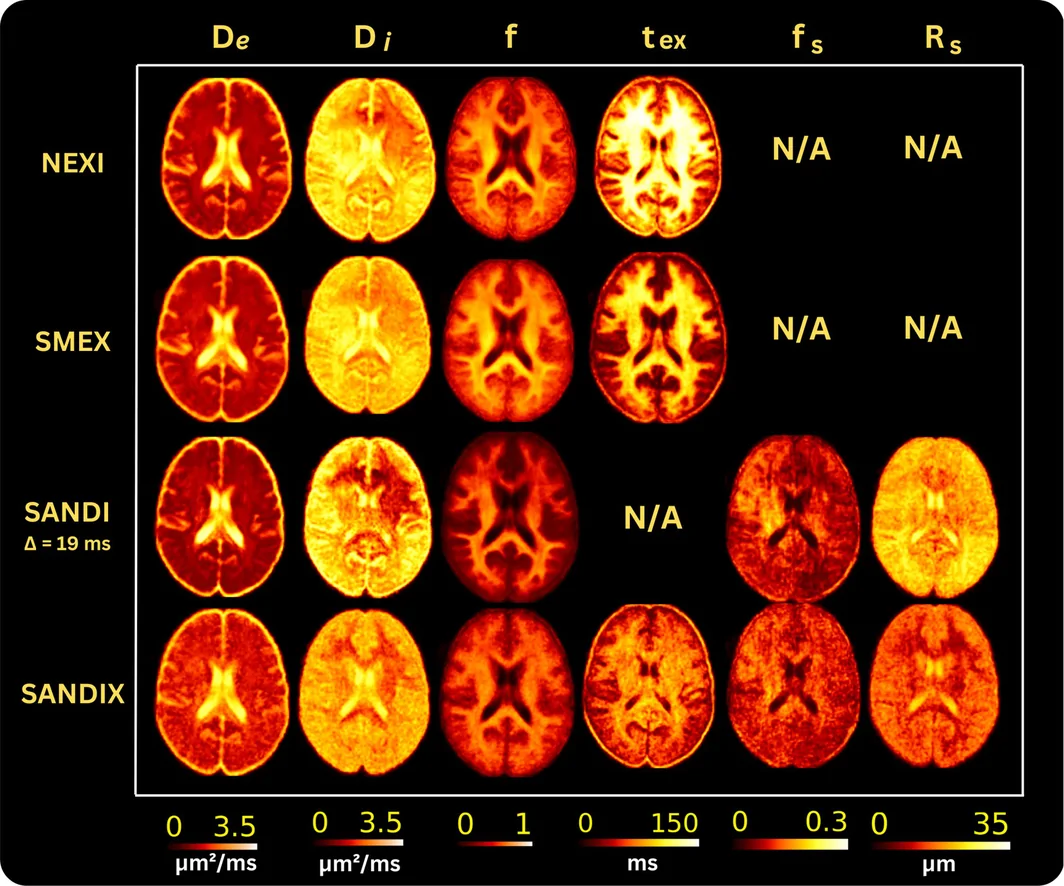
Average parameter maps across subjects in MNI space: axial slices of average parameter maps generated for each biophysical model; SANDI maps correspond to the Δ=19 ms acquisition, calculated across 26 subjects and represented in standard MNI space. The maps showcase spatial distributions of key model parameters, enabling a direct comparison of how different models characterize biophysical properties across the brain.

The unexpected asymmetry in $D_{i}$ and $f_{s}$ maps could be due to residual field gradient inhomogeneities that are captured by the model parameters with highest uncertainty. Individual subjects' powder‐averaged signal maps were visually inspected and showed these hemispheric differences, confirming that the parameter map asymmetry reflects data characteristics rather than fitting issue (Figure 4).

### 4.2 Focused Comparison on NEXI versus SMEX

#### 4.2.1 Simulation

**FIGURE 5:**
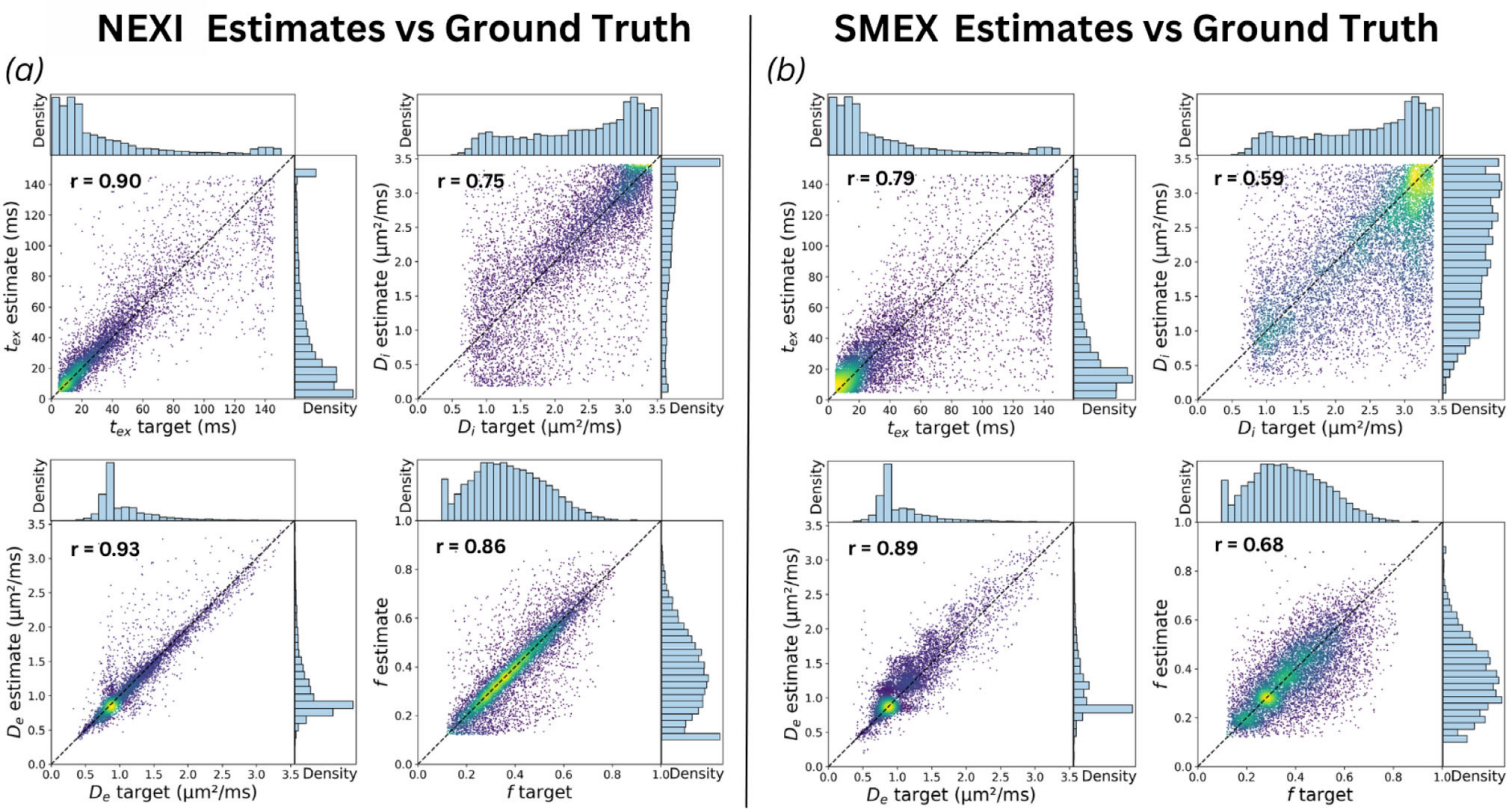
Comparison of parameter estimation from synthetic SMEX signals. Density‐colored scatter plots show estimated vs. ground truth parameters ($t_{\mathrm{ex}}$, $D_{i}$, $D_{e}$, f) for NEXI (a) and for SMEX (b). Synthetic ground truth SMEX signals were generated using the same δ, diffusion times, and b‐values as in vivo acquisitions. Signals were then powder‐averaged to emulate magnitude data. Histograms show marginal distributions; the dashed diagonal indicates perfect estimation. Pearson correlation coefficients (r) are displayed in each panel.

Estimation performance on the synthetic dataset is shown in Figure 5. $D_{e}$ and f were estimated with good accuracy and precision by both model variants, whereas $t_{\mathrm{ex}}$ was consistently underestimated and $D_{i}$ overestimated, also seen on previous reports using three diffusion times [11, 14]. The accuracy of $t_{\mathrm{ex}}$ further deteriorated for longer exchange times, reflecting the limited diffusion time range in a two‐Δ acquisition. Overall precision was poorest for both f and $D_{i}$.

Despite being fitted to the same noisy synthetic signals generated with SMEX, NEXI unexpectedly showed better overall estimation performance across parameters, while SMEX produced larger deviations, particularly for f, $D_{e}$, and $t_{\mathrm{ex}}$.

#### 4.2.2 Surface Maps for NEXI and SMEX

**FIGURE 6:**
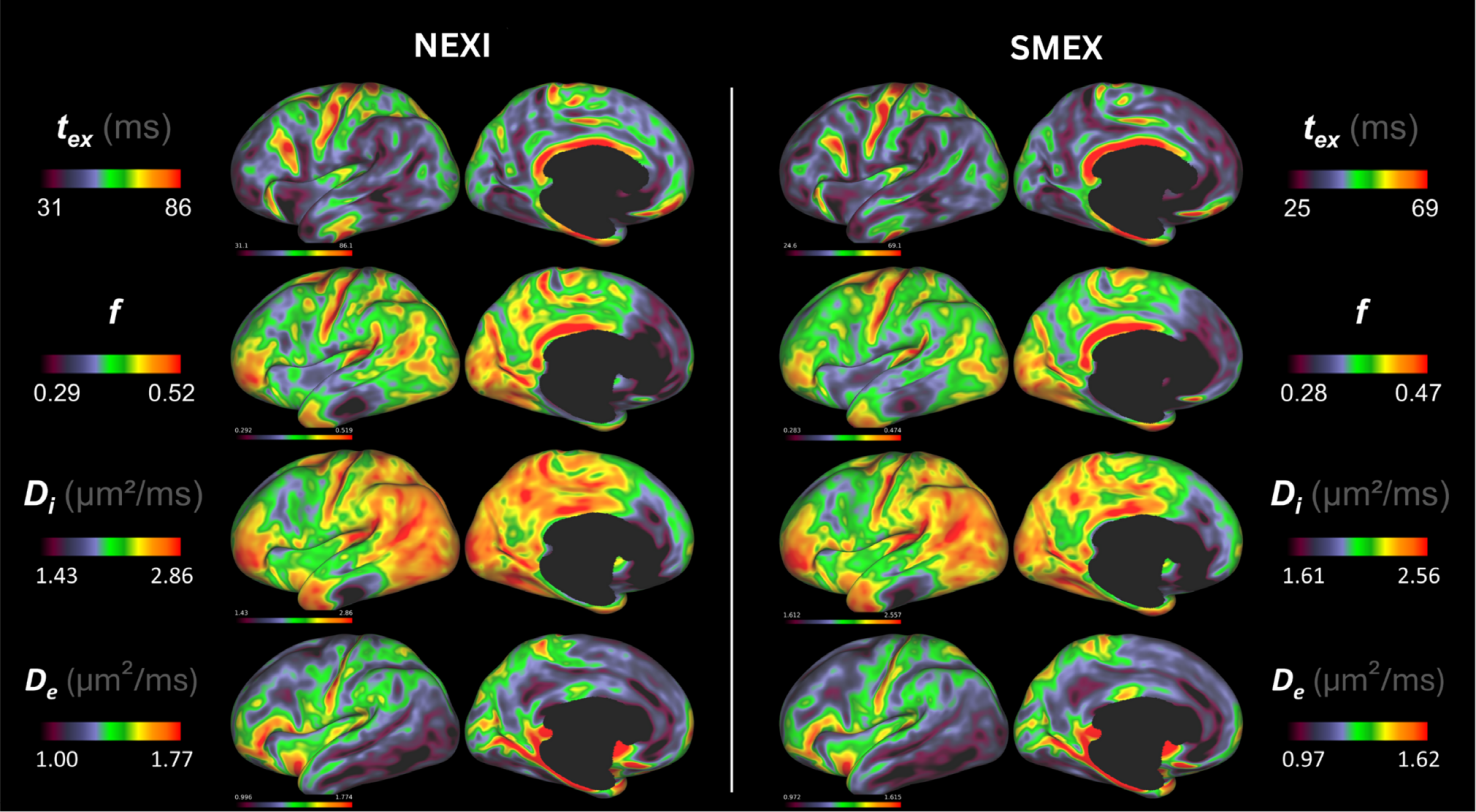
Projection onto cortical surface in fsaverage space of NEXI and SMEX, maps averaged across 25 subjects. Maps show a slight shift in the scales between models, but both show very similar anatomical trends.

Figure 6 shows human cortical surface maps for NEXI and SMEX estimated using two diffusion times. As expected, the two models show almost identical anatomical trends, with slight variations in their values and thus scales. SMEX shows lower $t_{\mathrm{ex}}$, f, $D_{e}$, and $D_{i}$ than NEXI, which is consistent with other works [11].

The estimated intercompartmental exchange time ($t_{\mathrm{ex}}$) exhibits elevated values in the somatosensory and motor cortices, including the precentral and paracentral regions, as well as in the occipital lobe and temporal lobe.

Similarly, the neurite volume fraction (f) demonstrates higher values in the occipital lobe. The relatively lower f observed in the anterior temporal lobe is also consistent with findings from NEXI studies conducted on clinical MRI systems [11].

Although the intra‐axonal diffusivity ($D_{i}$) map is the noisiest of the estimated parameters, it reveals some anatomical patterns that align with biological and model expectations. Specifically, $D_{i}$ appears to follow a similar spatial distribution to f, reflecting the association that regions with higher neurite density would result in faster intra‐neurite diffusivity.

Finally, the estimated extra‐axonal diffusivity ($D_{e}$) exhibits spatial distributions consistent with prior NEXI studies on both Connectom [14] and clinical scanners [11]. Notably, $D_{e}$ shows reduced values in the occipital lobe and elevated values in the somatosensory cortex. In addition, we observed faster $D_{e}$ in the insula, which was also reported previously [14].

## 5 Discussion

This analysis compared parameter estimates from four biophysical models using an open‐source CDMD dataset [12] to quantify different microstructural properties of cortical tissue in the human brain. Using the newly developed processing package “Gray Matter Swiss Knife”, we processed NEXI [7], SMEX [8], SANDIX [8] and SANDI [9] using two diffusion times instead of the four typically employed in earlier implementations of exchange models. This study serves as a proof of concept to assess whether these models can be reliably estimated from only two diffusion times. It compares parameter estimates across models, subjects, and previous studies, and assesses the quality of the model fits to synthetic and experimental data.

Previous NEXI papers have highlighted the importance of accounting for the Rician mean floor either in signal model or data processing for accurate fitting of NEXI parameters (particularly $t_{\mathrm{ex}}$) [11, 14]. Therefore, we investigated how two different denoising strategies impact parameter estimation across all four biophysical models: (1) Complex Denoising reduces Gaussian noise from the raw complex‐valued data to then retain the real‐valued signals in the fitting procedure; (2) Magnitude Denoising reduces noise in magnitude data and uses the concomitant noise variance estimation to correct for Rician bias in the model fit.

In addition, since NEXI and SMEX share the same underlying microstructural assumptions but differ in their mathematical and signal fitting formulations, we performed a focused comparison between them. Specifically, we evaluated (1) their accuracy in recovering synthetic ground truth signals using simulations, and (2) the anatomical plausibility of their cortical surface maps.

### 5.1 Cortical Ribbon Estimates

CDMD data fit showed that parameters common to all four models, namely f, $D_{i}$, and $D_{e}$, yielded cortical ribbon metrics in the ranges of f∼0.19–0.32, $D_{i}∼$2.2–3.0 ${\mathrm{\mu m}}^{2}/\mathrm{ms}$, and $D_{e}∼$0.79–1.09 ${\mathrm{\mu m}}^{2}/\mathrm{ms}$.

The estimated neurite fraction f was lower in SANDI and SANDIX compared to NEXI and SMEX. This likely reflects the explicit modeling of a soma compartment, which redistributes part of the intracellular signal from neurites to soma. As a result, the total intracellular fraction ($f+f_{s}$) remains within a similar range (∼20%–40%). This finding, however, contrasts with the common interpretation in NEXI and SMEX that the soma contribution is pooled into the extracellular compartment. Neurite fraction estimates for all models are in line with previous in vivo dMRI studies in both rodents and humans, which consistently report cortical GM neurite fractions in the range of 20%–40% [7, 8, 9, 11, 13, 14, 32, 33]. By contrast, histological studies often report higher neurite fractions, up to 60% [34], though this may reflect fixation‐induced shrinkage of the extracellular space in ex vivo tissue. Supporting this, ex vivo NEXI studies [35] report higher neurite fraction estimates, closer to the histological findings [8, 36].

Consistently across this and previous studies, $D_{i}$ was found to be the least robust parameter for all models, often exceeding $2.5\;{\mathrm{\mu m}}^{2}/\mathrm{ms}$ and frequently reaching the upper fitting bound. Estimated $D_{i}$ values across models and for both magnitude and real‐valued data ranged between 2.20 and 2.94 ${\mathrm{\mu m}}^{2}/\mathrm{ms}$, at times barely lower than the free water diffusivity at body temperature (3 ${\mathrm{\mu m}}^{2}/\mathrm{ms}$) [37]. Finally, parameter maps reveal substantial noise, underscoring the instability of this parameter, likely due to a flat fitting landscape along this dimension of parameter space [38, 39]. Among the tested models, SANDIX and SMEX yielded the most biologically plausible $D_{i}$ estimates, aligning better with axonal diffusivity values reported in WM [26, 40], with $D_{i}$ values ranging 2.20 to 2.46 ${\mathrm{\mu m}}^{2}/\mathrm{ms}$. It is noteworthy that both these models account for the actual pulse duration δ.

Previous works have shown that multi‐diffusion time datasets carry the signature of exchange rather than of soma restriction, in the form of lower signal with longer diffusion times, at a fixed b‐value [7, 8, 11]. Therefore, fitting SANDI to both diffusion times produces biologically implausible parameter estimates that diverge substantially from previous SANDI reports in human cortex (Table S1) and fails to properly capture time‐dependence across diffusion times (Figure 3). Therefore, consistent with the model's theoretical design, SANDI fitted using only the shortest diffusion time (Δ=19 ms) yields estimates more aligned with prior reports. Compared to fitting SANDI with two diffusion times (Table S1), the soma fraction in the cortical ribbon increased from 10% to 16%, and the estimated soma radius $R_{s}$ decreased from 24.3 to 9.5μm, which are much more aligned with other imaging literature [15, 32, 41]. A recent morphological study that analyzed 3500 GM cells estimated that neural soma sizes range from 2 to 30 μm in radius with an average of 7 μm [42]. Although still falling within the suggested range, there is a probable overestimation of the radius in these biophysical models, due to the volume weighting and the indirect non‐linear probe via diffusivity. A previous study estimated that this overestimation could be up to a factor of 1.6 for soma radii [8].

While SANDI parameter estimates were obtained using NLS within the Gray Matter Swiss Knife toolbox across all four models, we note important sensitivities to fitting algorithm choice. When fitting SANDI using RF implementation in CDMD data, soma fraction ($f_{s}$) maps display gray–white matter contrast consistent with previous literature (higher soma fraction in GM), whereas NLS produces maps with substantially reduced anatomical contrast (Figure 4). We report RF CDMD cortical ribbon estimates in Table S1 and show that compared to NLS, RF‐derived $f_{s}$ estimates were substantially higher and neurite fraction (f) was lower.

Despite this discrepancy, quantitative assessment of voxel‐wise residuals across the cortical ribbon demonstrated that NLS achieved lower residuals than RF across most b‐values, indicating superior data fit quality (Figure S4). This apparent paradox of better quantitative fit but less anatomical contrast between white and GM reflects known degeneracies in multi‐compartment diffusion models [38, 39, 43], where multiple parameter combinations can yield near‐identical signal predictions. NLS, by design, selects whichever combination minimizes residuals without regularization, and is highly sensitive to initialization location. In contrast, RF and AMICO methods incorporate implicit regularization (e.g., averaging across training examples, Tikhonov penalties) that biases solutions toward the training prior.

A concurrent study applying SANDI with NLS and RF on a different clinical dataset [11] reported identical findings: NLS demonstrated superior goodness of fit compared to RF, yet produced differences in volume fractions, supporting the conclusion that differences reflect fundamental differences in how these methods navigate the degenerate fitting landscape of multi‐compartment models. Given that the primary goal of this work is to compare four biophysical models (NEXI, SMEX, SANDI, SANDIX) using a consistent fitting framework, NLS was used across all models for fair model‐to‐model comparison. A comprehensive investigation of fitting algorithm effects is beyond the scope of this study, but deserves further investigation. Bayesian frameworks such as μGUIDE [44] that explicitly characterize posterior distributions and parameter identifiability may offer a path toward more transparent and reliable parameter estimates.

### 5.2 Quality of Fit

The goodness of fit analysis showed that NEXI was the best at fitting parameter estimates to powder‐averaged signal in the cortex. Similarly, SANDI fits suggest the exchange term is missing from the model, due to the challenge of capturing the correct time‐dependence. To address these limitations, more advanced GM models are currently being explored, such as SANDIX or the Generalized Exchange Model (GEM), which introduces a dedicated soma compartment along with exchange dynamics within the soma, aiming for improved biological accuracy.

Consideration of other components, like myelinated axons in GM, have also been previously proposed—enhanced SANDIX (eSANDIX) [8]. Although the signal contribution of myelinated axons is relatively small, it may become non‐negligible at high b‐values and could introduce subtle biases in parameter estimation if not modeled appropriately.

However, this increased biophysical realism comes at the cost of model complexity and a higher number of free parameters, which can hinder parameter stability. The poor fit performance of SANDIX reflects not simply the addition of parameters, but the fundamental challenge of simultaneously estimating several parameters—$t_{\mathrm{ex}}$, $f_{s}$, $R_{s}$—which have been shown to exhibit broad posterior distributions and high parameter degeneracy [30]. With six free parameters compared to four (NEXI/SMEX) or five (SANDI), the optimization landscape becomes increasingly complex, introducing greater parameter uncertainty and correlations that compromise fit precision.

Careful consideration is needed when extending biophysical models, especially when applied to in vivo data with inherent limitations in signal‐to‐noise and acquisition duration. Currently, the trade‐off between biological realism and model stability remains a central challenge in GM diffusion modeling. Further research into the contributions of diffusion signal from different components should be conducted to improve these models and balance specificity with precision.

### 5.3 Comparing NEXI versus SMEX

In this dataset, NEXI corrected for the Rician floor in the magnitude data yielded lower $t_{\mathrm{ex}}$ than NEXI without Rician mean correction (albeit on real‐valued data). The Rician mean correction has been shown to reduce estimation bias and stabilize $t_{\mathrm{ex}}$ given its high susceptibility to the noise floor [14]. A recent study [13] reported that estimation bias was reduced by a factor of ≈1.5 when using NEXI‐RM compared to ordinary NEXI. Moreover, it was shown that this correction had impact on the Connectom 1.0 scanner, but not on the Connectom 2.0, where the SNR was high enough for the Rician floor to be negligible.

Moreover, despite the use of phase images for complex denoising, residual noise still appears to influence the real‐valued data and the resulting $t_{\mathrm{ex}}$ over‐estimation, reaffirming the need for explicit noise modeling even in real‐valued fitting. It is therefore recommended to implement a Rician mean correction on magnitude data when fitting either NEXI or SMEX, unless ultra‐strong gradients that can yield high SNR images, such as those in Connectom 2.0, are available.

Surprisingly, NEXI performed better than SMEX in terms of goodness‐of‐fit to experimental data and accuracy and precision in simulations, while prior work had shown SMEX to outperform NEXI on synthetic signals. However, this difference is likely attributable to numerical implementation factors rather than fundamental model properties. Specifically, the solver used in SMEX relies on the numerical integration of a differential system via scipy.integrate.solve_ivp, which introduces additional imprecision—particularly problematic when high numerical precision is needed. In contrast, NEXI involves a direct integration using scipy.integrate.quad_vec, which tends to yield more stable results. Furthermore, in previous studies, the advantage of SMEX was shown when the NPA was indeed not met (e.g., with δ=16.5 ms), making NEXI less appropriate. In this work, with a shorter pulse duration (δ=8 ms), the assumptions behind NEXI are better met, and the numerical stability of its solver likely further contributes to its improved performance.

#### 5.3.1 Surface Maps

The anatomical patterns observed in our surface maps, particularly for parameters such as exchange time ($t_{\mathrm{ex}}$) and neurite fraction (f), are consistent with previously reported NEXI implementations and with known cytoarchitectonic and myeloarchitectonic cortical features [45]. Specifically, the elevated $t_{\mathrm{ex}}$, f, and $D_{i}$ values in primary sensorimotor cortices (including precentral, postcentral, and paracentral regions) align with the dense myelination and cell packing reported in classical histological and post mortem high field MRI studies [46]. These regions are known to contain large, heavily myelinated pyramidal cells (e.g., Betz cells in M1) [47], which likely contribute to the prolonged exchange times due to reduced permeability of their membranes.

The higher f observed in the occipital lobe, particularly in the primary visual cortex (V1, Brodmann area 17), is consistent with histological findings showing densely packed vertical bundles of apical dendrites [46, 48], as well as with previous imaging studies [9, 11, 14, 33]. Moreover, the relatively lower f observed in the anterior temporal lobe aligns with cytoarchitectonic descriptions of reduced cell density and laminar differentiation in this region [48], as well as with more recent parcellation studies based on microstructural imaging [49].

Furthermore, the surface map distribution of intra‐axonal diffusivity ($D_{i}$), although noisier, appears to follow similar patterns as f with a different scale, suggesting that regions with denser neurite packing naturally present faster intra‐neurite diffusivity. Indeed, higher neurite density may go in pair with more straight dendrite segments to reach a high packing, which in turn yields faster $D_{i}$. Meanwhile, extra‐axonal diffusivity ($D_{e}$) shows a distinct pattern with lower values in the occipital and temporal lobes and higher diffusivity in the somatosensory and insular cortices. These patterns have also been observed in prior NEXI studies using Connectom 1.0 protocols [14].

Together, these results highlight that the CDMD dataset‐derived parameter and surface maps reflect known biological distributions of cortical microstructure and are consistent across other NEXI, SMEX and SANDI implementations, and neuro‐anatomical patterns from other imaging modalities. Importantly, they support the feasibility of probing GM microstructure using only two diffusion times, offering a time‐efficient alternative to conventional protocols that typically require three or four diffusion times for NEXI estimation.

It is important though to highlight that, although the diffusion MRI protocol in this study used only two diffusion times, it included a total of 16 b‐values, 32 to 64 diffusion gradient directions depending on the b‐value, with a total scan duration of 55 min. While this is shorter than some high‐angular resolution protocols, this overall acquisition remains lengthy and limits feasibility in clinical environments due to its complexity.

Future work will focus on reducing scan time while preserving parameter sensitivity. Optimizing the combination of diffusion times, b‐values, and gradient directions could enable development of a more time‐efficient protocol, improving applicability in both research and clinical settings [50, 51, 52].

## 6 Conclusions

We retrospectively evaluated and compared the performance of four advanced biophysical models, NEXI, SMEX, SANDI and SANDIX, on a publicly available diffusion MRI dataset (CDMD) [12] fitted with the Gray Matter Swiss Knife Package.

These models varied in their goodness of fit against experimental data, with NEXI showing the best fit, followed by SMEX, and finally SANDIX.

The parameter estimates obtained in this study are consistent with those reported in previous studies. For exchange models such as NEXI and SMEX, we demonstrate that accurate and anatomically meaningful parameter estimates can be obtained using an acquisition protocol with only two diffusion times. Crucially, we emphasize the importance of explicitly modeling the noise, even when processing real‐valued data, as it significantly affects the exchange time parameter estimates ($t_{\mathrm{ex}}$). In the case of magnitude data, this translates into incorporating the Rician mean in the NEXI model, as previously introduced [14]. Additionally, we show that SANDI parameter estimates, particularly $f_{s}$, are sensitive to the choice of fitting algorithm, and that the model fails to capture multi‐diffusion time dependence due to the absence of an exchange compartment.

Future efforts should focus on further optimizing acquisition parameters to reduce the scan time and enable the clinical translation of GM models to patient populations, while ensuring interpretability and reliability of cortical microstructure estimates.

## Funding

This work was supported by the Swiss Secretariat for Education, Research and Innovation, MB22.00032 and the Swiss National Science Foundation, 194260.

## Supporting information


**Table S1:** Numerical values corresponding to Figure 2. Mean cortical ribbon parameter estimates with CI for each biophysical model. (A) Real‐valued data from 14 subjects. (B) Magnitude data with Rician mean correction from 24 to 25 subjects. (C) Previously reported estimates from the literature. SANDI results are shown for both diffusion times combined and for ∆ = 19 ms only.
**Figure S1:** CDMD acquisition parameters.
**Figure S2:** Distributions of voxel‐wise parameter estimates and DKT ROI‐wise medians in the cortical ribbon for NEXI and SMEX models, using magnitude data from the 26 subjects.
**Figure S3:** Quality of fit comparison between magnitude data (26 subjects) and real‐valued data (14 subjects). Dots represent experimental signal and curves show voxel‐wise model predictions averaged across the cortical ribbon. The figure highlights model accuracy and differences across diffusion times, *b*‐values, and data types. Goodness of fit was assessed using AICc, with lower values indicating better fit.
**Figure S4:** Voxel‐wise residual distributions for SANDI fitted with NLS and RF across the cortical ribbon (*n* = 11 665 960 voxels, 26 subjects). NLS achieves lower residuals than RF across most *b*‐values, indicating superior data fit quality despite producing parameter maps with reduced gray–white matter anatomical contrast.

## Data Availability

The CDMD dataset used in this study is publicly available at https://springernature.figshare.com/collections/_/5315474. The Gray Matter Swiss Knife toolbox used for model fitting is openly available at https://github.com/QuentinUhl/graymatter_swissknife. Code for model comparisons, statistical analyses, plotting, and cortical ribbon metric extraction can be made available upon request to the corresponding author.
