# Supplementary material for: Comparative Systematic Analysis of Gray Matter Biophysical Models on a Public Dataset
Source: Magn Reson Med. 2026 Jul 16;96(5):2278–91. doi: 10.1002/mrm.70507 (PMC13527255; doi:10.1002/mrm.70507)
Supplement: Supplementary file 1 [file MRM-96-2278-s001.pdf]

Supplementary Material

Supplementary Table 1 (Table S1): Numerical values corresponding to Figure 2. Mean cortical ribbon parameter estimates with CI for each biophysical model. (A) Real-valued data from 14 subjects. (B) Magnitude data with Rician mean correction from 24–25 subjects. (C) Previously reported estimates from the literature. SANDI results are shown for both diffusion times combined and for $\Delta = 19$ ms only.

**A: Real-valued data from 14 subjects**						
**MODEL**	t_{ex} (ms)	D_i (μm^2/ms)	D_e (μm^2/ms)	f	f_s	R_s (μm)
**NEXI**	**65.86** (53.60, 78.91)	**3.00** (2.74, 3.37)	**0.90** (0.86, 0.96)	**0.31** (0.28, 0.34)	-	-
**SMEX**	**39.94** (32.69, 48.21)	**2.41** (2.22, 2.62)	**0.99** (0.94, 1.09)	**0.36** (0.34, 0.39)	-	-
**SANDI 2td**	-	**2.95** (2.78, 3.19)	**0.78** (0.75, 0.86)	**0.19** (0.18, 0.22)	**0.09** (0.05, 0.15)	**24.85** (21.51, 27.42)
**SANDI ($\Delta = 19$ ms)**	-	**2.76** (2.19, 3.30)	**0.87** (0.82, 0.98)	**0.18** (0.16, 0.21)	**0.15** (0.13, 0.19)	**9.46** (9.12, 9.80)
**SANDIX**	**38.68** (35.42, 47.30)	**2.36** (2.16, 2.50)	**1.04** (0.97, 1.16)	**0.32** (0.31, 0.34)	**0.10** (0.08, 0.14)	**17.48** (15.35, 19.39)
**B: Magnitude Data from 24–25 subjects**						
**MODEL**	t_{ex} (ms)	D_i (μm^2/ms)	D_e (μm^2/ms)	f	f_s	R_s (μm)
**NEXI**$_{\text{RM}}$	**29.39** (21.21, 45.07)	**3.03** (2.73, 3.41)	**0.95** (0.88, 1.24)	**0.38** (0.34, 0.45)	-	-
**SMEX**$_{\text{RM}}$	**21.08** (17.97, 30.58)	**2.48** (2.29, 2.80)	**1.01** (0.91, 1.26)	**0.37** (0.34, 0.41)	-	-
**SANDI 2td**	-	**3.12** (2.95, 3.45)	**0.78** (0.75, 0.94)	**0.20** (0.18, 0.23)	**0.10** (0.05, 0.16)	**24.77** (21.81, 27.49)
**SANDI ($\Delta = 19$ ms)**	-	**2.94** (2.33, 3.40)	**0.89** (0.82, 1.10)	**0.18** (0.15, 0.21)	**0.16** (0.12, 0.20)	**9.49** (9.19, 10.21)
**SANDI ($\Delta = 19$ ms, RF)**	-	**1.77** (1.72, 1.82)	**1.30** (1.18, 1.54)	**0.11** (0.09, 0.15)	**0.42** (0.34, 0.46)	**8.47** (7.96, 8.67)
**SANDIX**	**26.15** (24.28, 31.06)	**2.21** (2.01, 2.37)	**1.09** (0.97, 1.44)	**0.30** (0.28, 0.32)	**0.09** (0.07, 0.13)	**17.29** (15.24, 19.69)
**C. Previous Papers**						
**MODEL**	t_{ex} (ms)	D_i (μm^2/ms)	D_e (μm^2/ms)	f	f_s	R_s (μm)
**NEXI**$_{\text{RM}}$ Connectome 1 (Uhl, 2024)	**42.3** [40.0, 44.7]	**3.35** [3.32, 3.38]	**0.92** [0.91, 0.93]	**0.38** [0.37, 0.38]	-	-
**NEXI**$_{\text{RM}}$ (Uhl, 2025)	**41.1** [15.5, 66.7]	**2.61** [2.03, 3.20]	**1.59** [1.18, 2.00]	**0.51** [0.41, 0.61]	-	-
**SMEX**$_{\text{RM}}$ (Uhl, 2025)	**36.8** [17.6, 58.0]	**2.45** [1.99, 2.90]	**1.23** [0.88, 1.61]	**0.42** [0.34, 0.50]	-	-
**NEXI** Connectome 1 (Chan, 2025)	**44.4** [30.2, 77.6]	**3.00** [3.00, 3.00]	**0.89** [0.81, 0.95]	**0.28** [0.27, 0.30]	-	-
**NEXI** Connectome 2 (Chan, 2025)	**20.8** [14.9, 33.5]	**3.00** [3.00, 3.00]	**0.88** [0.81, 0.94]	**0.34** [0.32, 0.34]	-	-
**SANDI ($\Delta = 39.07$ ms)** (Schiavi, 2023)	-	**2.45** [2.35, 2.55]	**1.36** [1.27, 1.45]	**0.34** [0.32, 0.36]	**0.26** [0.24, 0.29]	**12.7** [12.3, 13.1]
**SANDI ($\Delta = 19$ ms)** (Lee, 2024)	-	**1.83** [1.53, 2.13]	**1.60** [1.53, 1.67]	**0.17** [0.16, 0.18]	**0.42** [0.40, 0.44]	**9.26** [9.11, 9.41]

MGH Connectome Diffusion Microstructure Dataset (CDMD) Acquisition Protocol

Imaging Parameters:

- *Repetition Time (TR)*: **3800 ms.**
- *Echo Time (TE):* **77 ms.**
- *Field of View (FOV)*: **216 × 216 mm.**
- *Matrix Size*: **108 × 108.**
- *Slice Thickness:* **2 mm.**
- *Voxel Size:* **2 × 2 × 2 mm^3.**
- *Diffusion Times (Δ):* **19 ms or 49 ms.**
- *Diffusion-Encoding Gradient Duration (δ)*: **8 ms.**

For Δ = 19 ms:

- **B-values:**
  - 50 s/mm^2 - 32 directions
  - 350 s/mm^2 - 32 directions
  - 800 s/mm^2 - 32 directions
  - 1500 s/mm^2 - 32 directions
  - 2400 s/mm^2 - 64 directions
  - 3450 s/mm^2 - 64 directions
  - 4750 s/mm^2- 64 directions
  - 6000 s/mm^2 - 64 directions

For Δ = 49 ms:

- **B-values:**
  - 200 s/mm^2 -32 directions
  - 950 s/mm^2 - 32- directions
  - 2300 s/mm^2 - 32 directions
  - 4250 s/mm^2 - 64 directions
  - 6750 s/mm^2 - 64 directions
  - 9850 s/mm^2 - 64 directions
  - 13,500 s/mm^2 - 64 directions
  - 17,800 s/mm^2 - 64 directions
- **Diffusion Encoding Directions:**
  - 32 uniform directions for b-values < 2400 s/mm^2.
  - 64 uniform directions for b-values ≥ 2400 s/mm^2.

Supplementary Figure 1 (Figure S1): CDMD acquisition parameters.

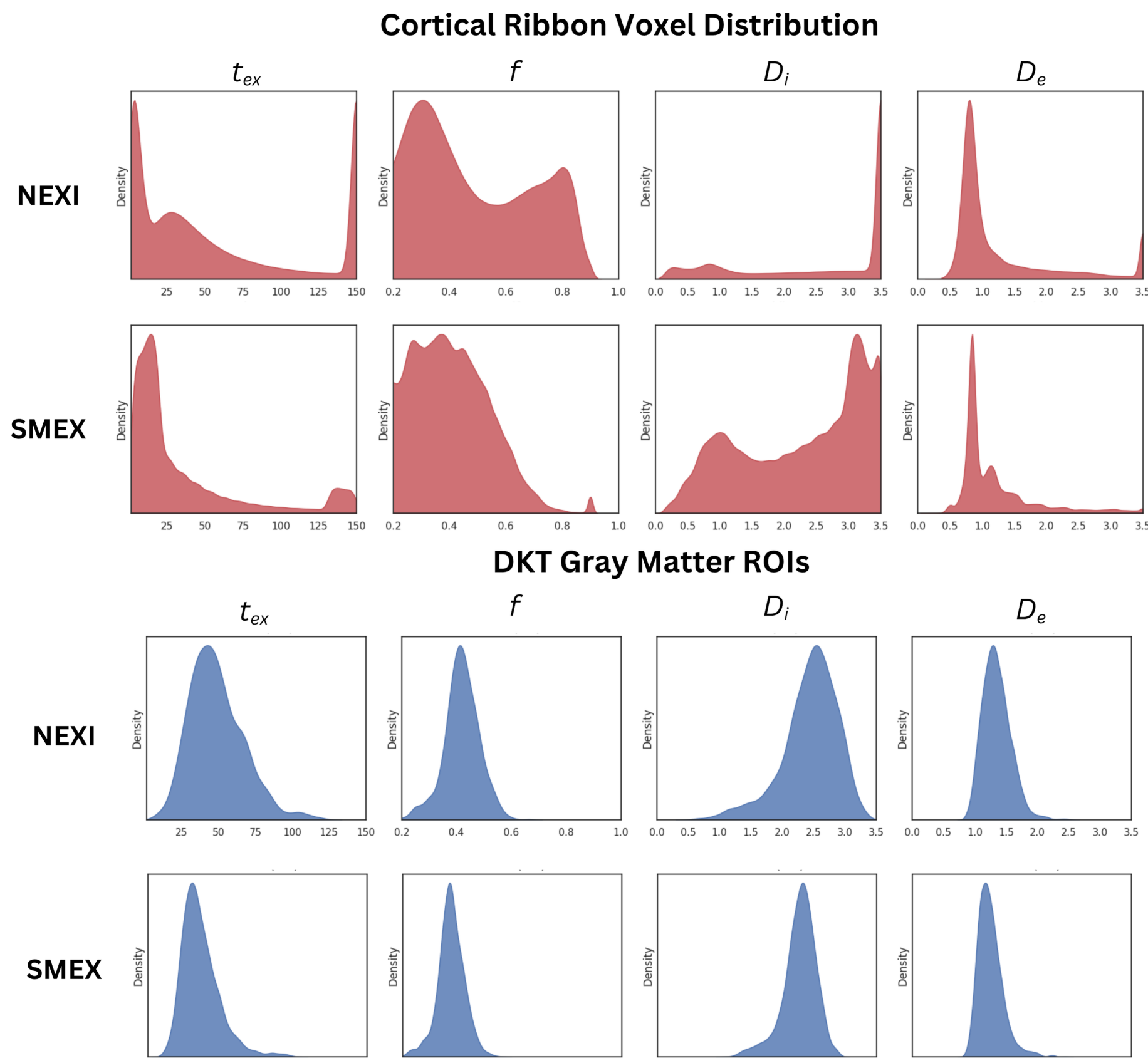

Supplementary Figure 2 (Figure S2): Distributions of voxel-wise parameter estimates and DKT ROI-wise medians in the cortical ribbon for NEXI and SMEX models, using magnitude data from the 26 subjects.

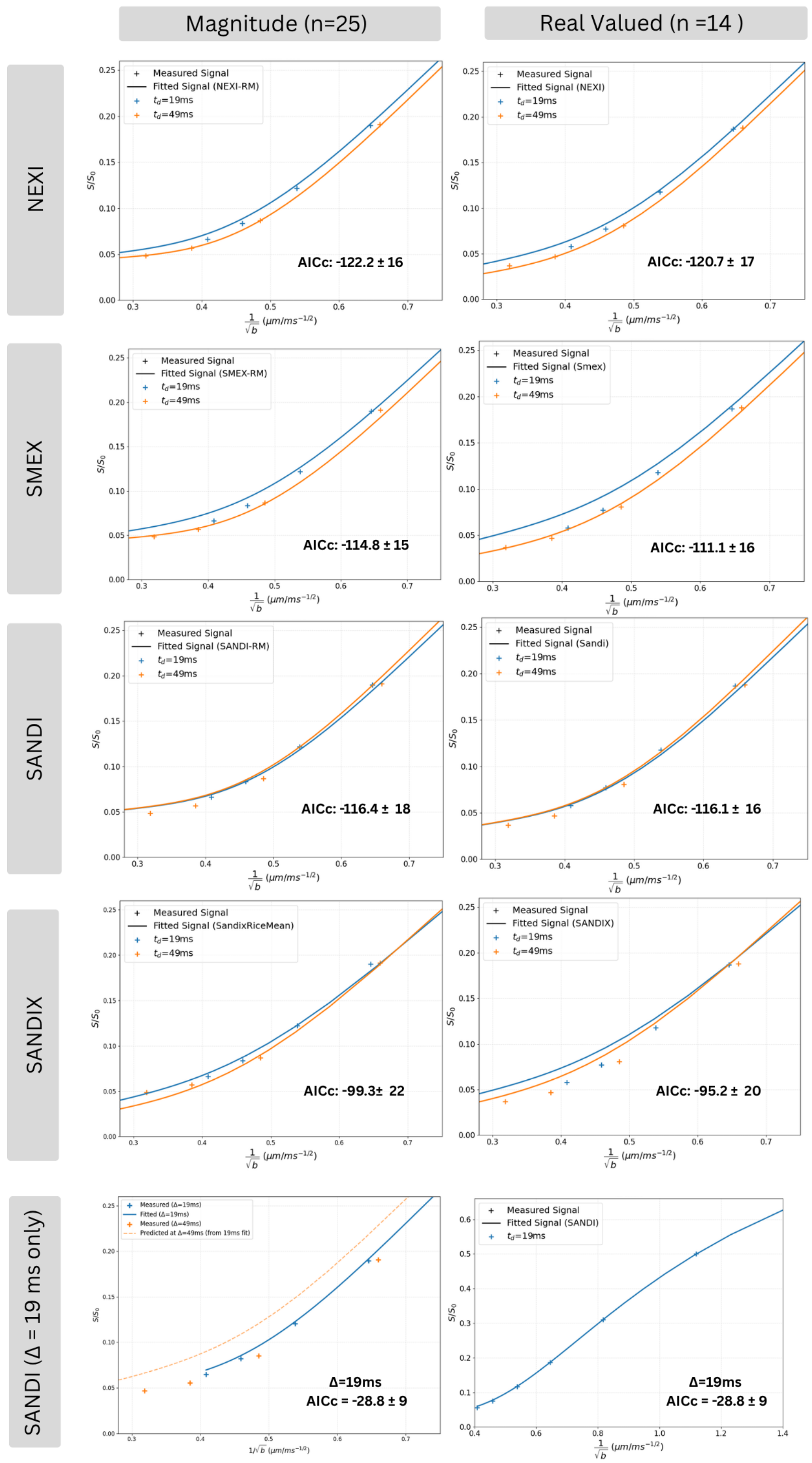

Supplementary Figure 3 (Figure S3): Quality of fit comparison between magnitude data (26 subjects) and real-valued data (14 subjects). Dots represent experimental signal and curves show voxel-wise model predictions averaged across the cortical ribbon. The figure highlights model accuracy and differences across diffusion times, b-values, and data types. Goodness of fit was assessed using AICc, with lower values indicating better fit.

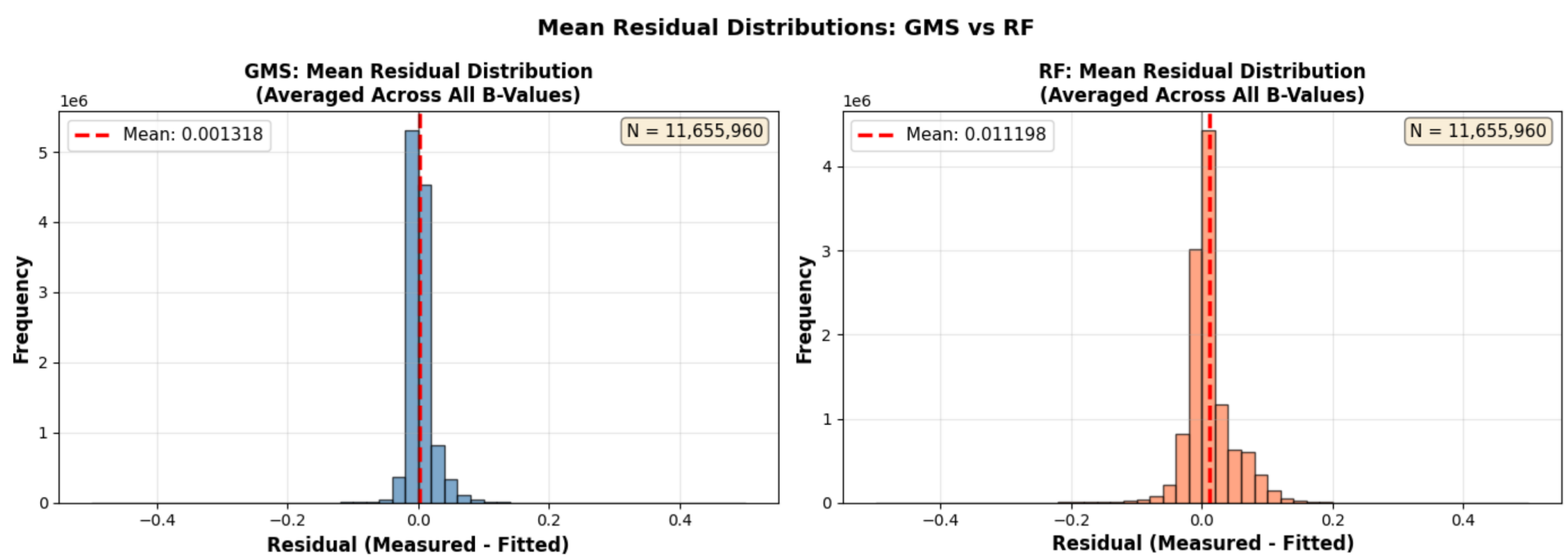

Supplementary Figure 4 (Figure S4): Voxel-wise residual distributions for SANDI fitted with NLS and RF across the cortical ribbon ($n = 11{,}665{,}960$ voxels, 26 subjects). NLS achieves lower residuals than RF across most b-values, indicating superior data fit quality despite producing parameter maps with reduced gray–white matter anatomical contrast.
